# Screening for Adult Attention-Deficit/Hyperactivity Disorder in a Psychiatric Outpatient Population with Specific Focus on Sex Differences

**DOI:** 10.3389/fpsyt.2017.00115

**Published:** 2017-06-30

**Authors:** Salvatore Corbisiero, Raffaela M. Hartmann-Schorro, Anita Riecher-Rössler, Rolf-Dieter Stieglitz

**Affiliations:** ^1^University of Basel Psychiatric Hospital, Clinical Psychology and Psychiatry, Basel, Switzerland; ^2^Department of Psychology, Division of Clinical Psychology and Psychiatry, University of Basel, Basel, Switzerland; ^3^University of Basel Psychiatric Hospital, Center for Gender Research and Early Detection, Basel, Switzerland

**Keywords:** attention-deficit/hyperactivity disorder in adults, screening, outpatients, sex differences, ASRS-v1.1

## Abstract

**Background/aims:**

Attention-deficit/hyperactivity disorder (ADHD) is often overlooked in adults; moreover, the problem seems to be even more critical in women. In the present, observational screening study, a clinical, particularly adult outpatient population was examined regarding frequency and severity of a likely ADHD, whereby sex differences were of particular interest.

**Methods:**

224 participants, 146 men and 78 women, were included. Based on data recorded with the self-rating WHO screening instrument *Adult ADHD Self-Report Scale* (ASRS-v1.1), it was examined how many participants were conspicuous for adult ADHD by exceeding a predefined cutoff value (COV) (COV ≥ 4 for ASRS-6, and ≥12 for ASRS-18). To examine frequency distributions, χ^2^ tests were conducted. For the inferential statistical comparison of means, *t*-tests for independent samples or Mann–Whitney *U* tests were calculated.

**Results:**

34.4% of the sample was screened positive in the ASRS-v1.1 screener short version, ASRS-6, while 17.4% were conspicuous in the symptom checklist, ASRS-18. There were indeed more men screened positive, but the difference in the frequency between the sexes was not statistically significant, indicating a balanced sex ratio. Further, severity of ADHD core symptoms inattention and hyperactivity/impulsivity was examined by comparing ASRS-18 symptom subscale scores. In concordance with the hypothesis, men and women did not differ in severity of symptoms.

**Conclusion:**

Results indicate that women might be affected by ADHD in a comparable manner as men; this emphasizes the importance for the awareness of ADHD in both sexes in clinical practice.

## Introduction

Attention-deficit/hyperactivity disorder (ADHD) is a neurodevelopmental disorder of multifactorial genesis that persists in 50–75% of cases into adulthood (1–4). Estimations of worldwide prevalence in adults range between 1.2 and 7.3% (5–7).

Attention-deficit/hyperactivity disorder manifests in the core symptoms inattention (IA), hyperactivity (HYP), and impulsivity (I); moreover, emotional dysregulation and disorganization are further prominent symptoms in adults (8). ADHD is classified according to the *International Statistical Classification of Diseases* [ICD-10 (9)] especially used in clinical practice, and the current, Fifth Edition of *Diagnostic and Statistical Manual of Mental Disorders* [DSM-V (10)], which is mainly applied in research.

Even though ADHD is a common disorder in adulthood, it is often overlooked (11, 12). An undetected ADHD can lead to severe psychiatric, functional and also forensic impairment (13). Moreover, patients concerned are often undergoing treatment due to other psychiatric disorders (11). Paying attention to comorbid disorders is important because they can mask symptoms of ADHD (14). Fayyad et al. (5) reported a seven times higher risk of having three or more disorders such as mood disorders, anxiety disorders, and substance use disorders when being affected by ADHD (OR = 7.2). ADHD patients are at a higher risk for delinquency (15); moreover, there is a problem of a higher distractibility in traffic which may lead to a higher involvement in accidents (16, 17). This raises the question how the disorder can be recognized adequately, since the process of recognizing and diagnosing is apparently hampered considerably by the comorbidity of other psychiatric disorders.

Screening is a method to identify disorders either before they manifest, when there are subclinical symptoms or when the disorder is already manifest but not yet detected. It is a pre-diagnostic method to estimate the occurrence and severity of symptoms (18); however, it is not a substitute to clinical diagnosis. The screening for a disorder is generally the first step of the diagnostic process. It initiates timely and appropriate diagnostic and treatment interventions preventing further suffering and impairments in major life activities for patients and reducing treatment costs. Screening is mostly conducted by the use of specific instruments, for example in the form of a self-rating questionnaire (18). A screening test shall separate healthy persons from affected ones. If a screening test reveals a positive result by achieving a predefined cutoff value (COV), a detailed evaluation of the psychiatric and somatic medical history of the patient, including physical, intellectual, and biographical development, clinical observation and differential diagnostics should follow (14). Specific diagnostic guidelines for ADHD in adults [see, e.g., Ref. (19)], a wide range of standardized and validated questionnaires and neuropsychological tests to objectify deficits of attention, working memory, and executive functions can facilitate and support the complex diagnostic process and the planning of further possible treatment (14).

Beyond the problem that ADHD in adults is often overlooked, recognizing the disorder adequately seems to be even more complex with specific regard to sex-related differences. Whereas there is a tremendous amount of research being conducted concerning ADHD in adults in general; however, findings regarding sex differences are inconsistent and new findings seem to be lacking. Most of research focuses on men, whereas women are often underrepresented or non-existent within study samples, particularly in clinical samples [e.g., Ref. (1, 20, 21)].

Regarding the prevalence of ADHD among men and women, ratios of approximately 3:1 were reported in population-based samples (21, 22). In a community-based sample with over 9,000 participants who were between 7 and 29 years old, Ramtekkar et al. (23) found male-to-female ratios of 2.09:1 for children, 2.56:1 for adolescents, and 2.15:1 for adults, respectively. Krause et al. (24) assumed an average male-to-female ratio of adult ADHD of 2–3:1 based on a clinical sample. Likewise, Fayyad et al. (5) and Kessler et al. (6) assumed that the prevalence of ADHD is higher in men than in women, specifying a male-to-female ratio of 1.6:1 (6).

Nevertheless, there are a few studies that also examined ADHD specifically in women [e.g., Ref. (20, 24, 25)]. Quinn (25), who called ADHD in women a *hidden disorder*, claimed that ADHD is often overlooked or misdiagnosed in women because their symptoms (e.g., forgetfulness, disorganization) are “less overt” (p. 579) than in men who show more often disruptive behaviors. For example, HYP in women shows up more often in a hyper talkative behavior instead of being physically active. Lauth and Raven (26) also assumed that the disorder is often overlooked in girls because they are less hyperactive and that symptoms in girls are not that obvious, e.g., at school (27). Differences in the manifestation and type of comorbid disorders make it even more complicated to recognize the disorder adequately in women (25).

Grevet et al. (28) and Rasmussen and Levander (29) found that men and women did not differ in symptom manifestation and severity. Biederman et al. (30) likewise did not find sex differences in the frequency of subtypes, whereby the combined subtype was the most frequent among both sexes, followed by the inattentive and the hyperactive-impulsive subtype. Ramtekkar et al. (23) determined that the inattentive subtype was the most frequent and the hyperactive–impulsive the least frequent subtype in both sexes. These findings are consistent with Krause et al. (24) who likewise did not find sex differences among ADHD subtypes in adults. Retz-Junginger et al. (31) found, when using self-report instruments, sex differences in I but not in IA and HYP. In the same study, they found sex differences in IA but not in HYP and I when applying semi-standardized interviews (31). Although there were certain sex differences between the examined ADHD patients, Retz-Junginger et al. (31) concluded that the found differences in core symptoms were not particularly prominent. However, it is important to take into account that methodological issues, such as selection of instruments (31), rater biases, comorbid disorders, referral source (21), and small sample sizes (27), may influence results of given findings.

The present, observational study was designed to record ADHD symptoms in adults in a clinical population. To our knowledge, there has not been done any research concerning the examination of sex differences in ADHD on the level of screening tests. Therefore, the following research questions are examined.

First, it was analyzed how many participants within the population report conspicuous ADHD symptoms on a validated screening questionnaire, being consequently likely to have an adult ADHD. The frequency of conspicuous participants was compared to the frequency of inconspicuous participants. Second, with regard to sex, it was examined how many men and women were conspicuous for ADHD. Third, men and women were analyzed regarding the severity of the core symptoms IA and HYP/I by comparing conspicuous symptom scores of the screening questionnaire. Considering the current state of research, it was hypothesized that there are significant more men than women with conspicuous ADHD symptoms. Given the heterogeneous findings of existing literature, it was rarely possible to make an assumption about sex differences regarding symptom severity across ADHD core symptoms in the examined population. However, because the majority of the recent findings about sex differences in adults did not find significant differences, it was predicted that men and women do not differ in symptom severity assessed by the screening questionnaire.

## Materials and Methods

### Participants

The sample included a total of 224 patients who were recruited across eight, mainly outpatient clinics that belong to the *University of Basel Psychiatric Clinics* (UPC) in Basel, Switzerland. Patients in these eight clinics usually do not undergo an ADHD screening. Only for this specific study these clinics screened patients. Exclusion criteria were first of all an insufficient command of German, an intelligence quotient of IQ < 85, schizophrenia or other psychotic disorders, a current or most recent episode of a manic or current severe major depressive disorder, acute stress disorder, or substance intoxication and withdrawal. Participants’ characteristics are presented in Table 1. There were 146 (65.2%) men and 78 (34.8%) women, which is approximately equivalent to a male-to-female ratio of 2:1 with a statistical difference [χ^2^(1, *N* = 224) = 20.64, *p* < 0.001]. Two participants were excluded due to the amount of missing answers (>20%). The age of the participants ranged from 18 to 75 years (*M* = 36.05, *SD* = 11.45), whereby men and women did not differ in average age [*t*(222) = 1.09, *p* = 0.276]. All participant were attended in one of the eight clinics at UPC with group differences in gender [χ^2^(7, *N* = 243) = 18.07, *p* = 0.012] and age [ANOVA: *F*(7, 224) = 8.44, *p* < 0.001]. Participants gave written informed consent. The *Ethics Committee of Basel* (Ethikkommission beider Basel) approved the study (reference number: 384/08).

**Table 1:** Characteristics of participants (*N* = 224) and clinics of registration.

	*M* (*SD*) or absolute number (%)
**Sex**
Male: female	146 (65.2%): 78 (34.8%)***
**Age (years)**
Male: female	36.66 (11.01): 34.91 (12.22)^+^
Overall	36.05 (11.45)
Range	18.00–75.00
**Clinic of registration**
ADS	84 (37.5%)
VTA	46 (20.5%)
ZA	38 (17.0%)
ZDK	20 (8.9%)
ZASS	13 (5.8%)
ZFM	10 (4.5%)
U3	8 (3.6%)
FAM	5 (2.2%)
Total	224 (100.0%)

p, p-values for sex obtained by χ^2^ test (***p < 0.001) and age by unpaired t-test; clinic of registration, clinic where the participants completed the questionnaires; ADS, Ambulance Addiction Services (Ambulanz für Sucht); VTA, Ambulance Behavioral Therapy (Verhaltenstherapie Ambulanz); ZA, Central Patient Admission (Zentrale Aufnahme); ZDK, Centre for Diagnostic and Crisis Intervention (Zentrum für Diagnostik und Krisenintervention); ZASS, Center for Affective, Stress and Sleep Disorder (Zentrum für Affektive-, Stress- und Schlafstörungen); ZFM, Centre for Psychotic Disorders and Transcultural Psychiatry (Zentrum für psychotische Erkrankungen und transkulturelle Psychiatrie); U3, Inpatient Unit for Substance Dependence and Addiction (Stationäre Abteilung für Abhängigkeit und Sucht); FAM, Forensic Ambulance (Forensische Ambulanz).

***p < 0.001.

*^+^p = 0.276*.

### Procedure

The study was conducted between March 2013 and August 2014. In order to collect the data about adult ADHD, ASRS-v1.1 (32, 33) was distributed to eight, particularly outpatient clinics at UPC (see Table 1). The employees of the respective clinic, first of all psychiatrists and psychologists, passed the questionnaire to the participants and obtained written informed consent. The instruction how it needed to be completed was provided on the questionnaire itself. The questionnaires were regularly collected, evaluated, and sent back to the attending psychiatrists and psychologists, accompanied by a recommendation of diagnostics and/or treatment. Patients who exceeded the COV were recommended for a specific ADHD consultation which is offered by the ADHD Special Consultation of the Outpatient Department at UPC.

### Instruments

*Adult ADHD Self-Report Scale* [ASRS-v1.1 (32, 33)]: The self-rating scale is based on the 18 DSM-IV ADHD criteria and may provide information suggesting the need of a more in-depth diagnostic evaluation. Six (part A) of the 18 questions were found to be the most predictive of symptoms consistent with ADHD in adulthood (33). The 12 remaining questions form part B of the checklist providing additional cues on the patient’s symptoms. The instrument, with its five-point scale from never (0) to very often (4), takes about 5 min to complete. The total score is calculated by summing the values of all items. The higher the score is the more symptoms are pronounced. In addition to the sum score of the checklist, the two subscales IA and HYP/I can be calculated. While part A helps to identify patients correctly, part B records the severity of symptoms. Part A is seen as the short version of the checklist [ASRS-6 (33)]. This consists of four items from the IA and two items of the HYP*/*I subscale. By reaching a COV of 4 in ASRS-6, an adult ADHD is likely. This COV is reached, when an individual exceed a certain value in order for the symptom to be counted (all symptom-ratings exceeding 1 or 2 on the five-point scale). Kessler et al. (33) were able to establish good psychometric properties for the screener (ASRS-6) and the full version (ASRS-18): sensitivity: 68.7 versus 56.3%, specificity: 99.5 versus 98.3%, total classification accuracy: 97.9 versus 96.2%, and Cohen’s κ: 0.76 versus 0.58. Buchli-Kammermann et al. (11) determined the psychometric quality of the German version. The authors determined similar values for both versions (ASRS-6 versus ASRS-18): sensitivity: 66.6 versus 72.3%; specificity: 64.9 versus 68.1%, Cronbach’s α: 0.73 versus 0.89. For the screening process, the ASRS-6 proved to be particularly relevant. Ramos-Quiroga et al. (34) revealed a new strategy in scoring symptoms by using a quantitative ranking between 0 and 24 points for either subscale (IA and HYP*/*I) with a COV at 12 points (sensitivity: 96.7%, specificity: 91.1%, Cohen’s κ: 0.88). Yeh et al. (35) who also recently examined the ASRS-v1.1 likewise used a quantitative ranking by classifying patients with a sum score of 24 on either subscale having highly likely ADHD, patients with scores of 17–23 of likely and those with scores of 0–16 of unlikely having ADHD. For the current study the short version (ASRS-6) as well as the full version (ASRS-18) of this screening instrument was used. The COV of 12 points in the full version (ASRS-18) proposed by Ramos-Quiroga et al. (34) was adopted.

### Data Analysis

For the data analyses, IBM SPSS Statistics version 21 was used. First, the sample was described regarding probable differences in gender, age, and clinics. To examine frequency distributions, χ^2^ tests were conducted. For the inferential statistical comparison of means, *t*-tests for independent samples were calculated. In cases where distribution requirements were violated, Mann–Whitney *U* tests were conducted.

## Results

### Frequency of Positive ADHD Screenings in the Overall Sample

Overall, there were significantly more participants who did not exceed the COVs in ASRS-6 and ASRS-18 than participants who exceeded the COVs. 34.4% of the participants screened above and 65.6% below the COV of ASRS-6. In ASRS-18, 17.4% of the participants screened above and 82.6% below the COV. The percentage of participants who exceeded the COV in ASRS-6 differed from the percentage of those who exceeded the COV in ASRS-18 [χ^2^2(1, *N* = 224) = 64.17, *p* < 0.001]. These results are presented in Table 2.

**Table 2:** ASRS-v1.1 COVs of the overall sample (*N* = 224) and separated by sex.

	Overall	Sex			
		Male	Female		
				
	*n* (%)	*n* (%)	*n* (%)	χ^2^ (*df*)	*p*-Value
**ASRS-6** COV (≥4)				2.02 (1)	0.155
Exceeded	77 (34.4)	55 (37.7)	22 (28.2)		
Not exceeded	147 (65.6)	91 (62.3)	56 (71.8)	21.86 (1)	<0.001*

**ASRS-18** COV (≥12)				1.75 (1)	0.185
Exceeded	39 (17.4)	29 (19.9)	10 (12.8)		
Not exceeded	185 (82.6)	117 (80.1)	68 (87.2)	95.16 (1)	<0.001*

*The percentage of participants who exceeded COVs differed from the percentage of those who did not exceed COVs (*p < 0.05).

*χ^2^, chi-square; *df*, degrees of freedom; *p, p*-values. ASRS-6, *Adult* Attention-Deficit/Hyperactivity Disorder (*ADHD) Self-Report Scale*, ASRS-v1.1 screener; ASRS-18, *Adult ADHD Self-Report Scale*, ASRS-v1.1 symptom checklist; COV, cutoff value*.

### Frequency of Positive ADHD Screenings in Men and Women

Of the 146 men, 55 exceeded the COV in ASRS-6 and 29 in ASRS-18. Of the 78 women, 22 exceeded the COV in ASRS-6 and 10 in ASRS-18. This means that 37.7% of all men within the sample exceeded the COV in ASRS-6 and 19.9% in ASRS-18. 28.2% of all women exceeded the COV in ASRS-6 and 12.8% in ASRS-18. Hence, there were more men with a likely ADHD in the study population; however, the belonging to either sex group did not have a significant impact of exceeding the COV, neither in ASRS-6 [χ^2^2(1, *N* = 224) = 2.02, *p* = 0.155] nor in ASRS-18 [χ^2^2(1, *N* = 224) = 1.75, *p* = 0.185]. These results are also presented in Table 2.

### Sex-Specific Symptom Severity

*M*s and *SD*s of the participants’ achieved symptom scores that were of relevance for the interpretation of the following statistical tests, are presented in Table 3. To examine whether men and women of the overall sample differ regarding symptom severity, two-tailed *t*-tests for independent samples were conducted. Among the 224 participants, men had slightly higher symptom scores on both subscales; however, the differences were not significant, neither on subscale IA [*t*(222) = 1.03, *p* = 0.305] nor on subscale HYP/I [*t*(222) = 1.48, *p* = 0.140].

**Table 3:** Descriptive statistics of ASRS-v1.1 subscales IA and HYP/I by COV and sex.

	Subscale	Sex	*n*	*M*	*SD*	*p*-Value
**Overall**					
	IA	Male	146	16.88	7.48	0.305
		Female	78	15.85	6.64	
	HYP/I	Male	146	15.86	6.91	0.140
		Female	78	14.45	6.52	
**ASRS-6** COV (≥4)						
Exceeded	IA	Male	55	23.24	4.99	0.775
		Female	22	22.86	5.53	
	HYP/I	Male	55	20.65	5.69	0.109
		Female	22	18.32	5.74	
Not exceeded	IA	Male	91	13.04	5.97	
		Female	56	13.09	4.75	
	HYP/I	Male	91	12.96	5.91	
		Female	56	12.93	6.21	
**ASRS-18** COV (≥12)						
Exceeded	IA	Male	29	25.97	4.73	0.984
		Female	10	26.00	4.90	
	HYP/I	Male	29	24.14	4.30	0.301
		Female	10	22.40	5.15	
Not exceeded	IA	Male	117	14.63	6.23	
		Female	68	14.35	5.46	
	HYP/I	Male	117	13.80	5.82	
		Female	68	13.28	5.87	

*IA, inattention; HYP/I, hyperactivity/impulsivity; ASRS-6, *Adult* Attention-Deficit/Hyperactivity Disorder (*ADHD) Self-Report Scale*, ASRS-v1.1 screener; ASRS-18, *Adult ADHD Self-Report Scale*, ASRS-v1.1 symptom checklist; COV, cutoff value*.

Among the participants who were conspicuous for ADHD in ASRS-6, men also had higher symptom scores, but again, the differences between men and women on subscale IA [*t*(75) = 0.29, *p* = 0.775] and subscale HYP/I [*t*(75) = 1.62, *p* = 0.109] were not significant. Among the participants who were conspicuous for ADHD in ASRS-18, women had slightly higher scores on subscale IA, while men had higher scores on subscale HYP/I. However, there were likewise no significant differences between men and women each on subscale IA [*t*(37) = 0.02, *p* = 0.984] and subscale HYP/I [*t*(37) = 1.05, *p* = 0.301].

Because the assumption of normality distribution was violated among the sexes, Mann–Whitney *U* tests were conducted. Among the 224 participants, average symptom scores on subscale IA [*U*(78,146) = 5,093, *p* = 0.193] and HYP/I [*U*(78,146) = 5,050.5, *p* = 0.163] did not differ between the sexes. There were likewise no significant differences between men and women among those participants above the COV in ASRS-6 for IA [*U*(22,55) = 597, *p* = 0.928] and HYP/I [*U*(22,55) = 457, *p* = 0.094] and those above the COV in ASRS-18 for IA [*U*(10,29) = 139, *p* = 0.846] and HYP/I [*U*(10,29) = 132, *p* = 0.674]. As predicted, men and women did not differ in the core symptomatology calculated for both symptom subscales, IA and HYP/I.

## Discussion

The present study was designed, first, to examine how many participants are likely to have an adult ADHD by achieving a conspicuous COV in the screening questionnaire ASRS-v1.1. Second, it was of interest how many men and women are likely to have an ADHD, hypothesizing that there are significantly more men with a likely ADHD than women. And third, men and women were examined regarding differences in the severity of ADHD core symptoms IA and HYP/I assessed by ASRS-18, predicting that there are no significant differences in severity between the sexes.

It is noteworthy that with 34.4% of participants who likely have an ADHD according to ASRS-6 and 17.4% according to ASRS-18, respectively, the rates obviously differ (see Table 2). Within this framework, there is no final explanation for this difference. The quality of psychometric criteria of both ASRS-v1.1 versions were recently examined by Konstenius et al. (36) on a female offenders sample, and they also found different prevalence rates in ASRS-6 and ASRS-18. However, they found that both versions had a convincing sensitivity of 100%, but that ASRS-18 had a lower specificity (45 versus 66%) and a lower positive predictive value (42 versus 55%) than ASRS-6. Either way, symptom subscales scores from ASRS-18 were finally useful for the present study because they revealed more detailed information about symptom severity, assessing for example symptoms of I that were not considered in ASRS-6. The findings of the present study support previous findings which indicated that ADHD is a frequent disorder in adults (5, 6, 37), emphasizing the importance of screening and the initiation of further diagnostics in case of a positive screening result.

In contrast to our prediction about the frequency of a suspected ADHD in men and women, there were namely more men than women who exceeded the COV of ASRS-6 and/or ASRS-18; however, the difference in frequency between the sexes was not statistically significant. Nevertheless, this finding is meaningful, as it shows that the percentage of men screened positive for ADHD is indeed higher than in women, but the difference is not that large as supposed by a majority of other studies, particularly studies with children samples [e.g., Ref. (21, 38)], indicating that adult women are affected almost as often as men. This finding is supported by the assumption that sex ratios are more balanced in adults than in children (24).

In concordance with our prediction, men and women did not differ in the severity of the core symptoms IA and HYP/I. This finding is supported by other empiric studies which assumed that there are no or only few differences between men and women concerning the core symptomatology [e.g., Ref. (28, 29, 31, 39)]. Retz-Junginger et al. (31) examined sex differences with different types of instruments and revealed inconsistent results. For self-reports, they indeed found only some sex differences in I, concluding that male and female ADHD patients seem to be “more similar than different and that symptoms of ADHD might not be sex-specific” [(31), pp. 100]. However, the study of Robison et al. (40) presented to some extent other. In this study comparing the attributes of men and women in a large, controlled ADHD study, there were significant differences regarding the type of ADHD. Women were more often given a combined ADHD diagnosis that men and men had a higher frequency of inattentive subtype compared to women.

This study bears theoretical and practical implications. This is, apparently, one of the first studies that examined sex differences on the base of an ADHD self-report screening questionnaire. It was previously shown that exceeded COVs on both ASRS-v1.1 versions, ASRS-6 and ASRS-18, are good predictors for adult ADHD (11, 33–35). Therefore, it might be important and helpful to screen patients who are undergoing psychiatric treatment due to other disorders preventively and routinely for ADHD.

This study contributes to the sensitization to ADHD in adults, particularly to screening ADHD in women. With 28.2% women screened positive in ASRS-6 and 12.8% in ASRS-18, it seems to be obvious that ADHD in women is not a hidden disorder as Quinn (25) claimed. In the contrary, although the number of positively screened men exceeds the one of positively screened women, the results indicate that men with ADHD seem not to be a significant majority. Furthermore, both sexes seem to be comparably impaired by ADHD, as shown, for example, in Retz-Junginger et al. (39). Robison et al. (40) could even observe that women with ADHD are more impaired than men and show a more complicated presentation of the disorder with greater rates of affective or emotional symptoms. They had higher level of ADHD symptomatology, had more symptoms of depression and anxiety and were more likely to have problems with sleep and somatic complaints.

It is important to acknowledge some limitations. Since this is a screening study without further diagnostic assessment, the results should be interpreted against the background of a tendency which can be put in relation to other studies. However, the findings about frequency and severity are not to be directly applied to the findings of studies which were made with diagnosed ADHD patients. Moreover, due to the fact that the study design is clinical and observational, there is no healthy control group. This might restrict the generalization to non-clinical populations. Besides, participants were not randomized to the different clinics. Having an equal amount of participants in each clinic would eliminate the impact of this variable and strengthen the explanatory power of the variables of interest.

There are also several risks of bias. The present data base on self-reports. It was found that adults tend to underestimate their ADHD symptoms (6, 41). Therefore, answers could be biased to some extent. Moreover, a more balanced sex ratio in the overall sample was desirable, but, given existing literature, the present male-to-female ratio of approximately 2:1 is often seen in clinical populations [e.g., in Ref. (31)] and can be, therefore, considered as representative within this type of population. In order to make statements about general population in future, a more balanced sex proportion will be necessary.

It will be important to replicate this study by extending the method of assessing ADHD symptoms in a randomized, controlled trial with more participants. Namely, there are symptoms beyond the core symptomatology that are estimated to be particularly frequent in females such as emotional dysregulation (40). Examples of ADHD self-report instruments that were shown to have robust psychometric criteria and validity recently (42, 43) and that assess ADHD typical symptoms beyond ASRS-v1.1, such as emotional dysregulation, are the *Conners’ Adult ADHS Rating Scales* (44) and *Wender-Reimherr Adult Attention Deficit Disorder Rating Scale* (42, 45). Retz-Junginger et al. (39) and Robison et al. (40) found sex differences in emotional dysregulation with women having higher scores when applied UTAH criteria (45, 46). However, further results of these two studies are contradictory. While Retz-Junginger et al. (39) did not find sex differences in severity of the core symptomatology of ADHD according to DSM-IV-TR, Robison et al. (40) could show that women have more symptoms of IA and disorganization than men.

Further, the assessment and analysis of other psychiatric and comorbid disorders of the participants is highly recommendable in order to make more accurate statements regarding ADHD, gender differences, and its comorbid psychiatric disorders bearing in mind the previous mentioned risk of overlooking ADHD in adults (11). Recent findings by Park et al. (47) who assessed ADHD symptoms by ASRS-6 and examined further comorbid disorders in an epidemiological Korean study showed that ADHD was highly correlated with somatoform disorders, sleep disorders, mood disorders, suicidality, anxiety disorders, and substance abuse. Given the elevated risk of comorbid disorders (5, 14), it is possible that women and men differ in type and frequency of comorbid disorders (29). There are findings that assume for example mood disorders, anxiety disorders, and personality disorders to be more frequent in women with ADHD than in men with ADHD (48). The clinics of registration may have a significant impact on the analysis of interest. Since there is a broad range of psychiatric disorders treated in the participating clinics, this recalls the problem stated in the introduction that ADHD can be masked through other psychiatric and comorbid disorders and remains undetected without screening (14). Finally, another aspect should be considered in further studies: Other psychiatric disorders might mimic ADHD symptoms, leading to false-positive screening of ADHD. To further understand the gender distribution in ADHD in a clinical sample, it is mandatory to consider all these mentioned points.

## Conclusion

Screening for ADHD is a useful and reliable method to detect adult ADHD in a clinical population, a disorder that is of important relevance in men and women and that should not be overlooked. ADHD in women is as common as in men; therefore, it deserves more attention in women. Screening for ADHD can guide subsequent diagnostic follow-up assessments with the establishment of appropriate treatment plans which can have a positive impact on the overall treatment success.

## Ethics Statement

This study was carried out in accordance with the recommendations of *Ethics Committee of Basel* (Ethikkommission beider Basel, EKBB) with written informed consent from all subjects. All subjects gave written informed consent in accordance with the Declaration of Helsinki. The protocol was approved by the *Ethics Committee of Basel* (Ethikkommission beider Basel, EKBB).

## Author Contributions

All authors contributed extensively to the work presented in this paper.

## Conflict of Interest Statement

The authors declare that the research was conducted in the absence of any commercial or financial relationships that could be construed as a potential conflict of interest.
